# Time-lapse imagery and volunteer classifications from the Zooniverse Penguin Watch project

**DOI:** 10.1038/sdata.2018.124

**Published:** 2018-06-26

**Authors:** Fiona M. Jones, Campbell Allen, Carlos Arteta, Joan Arthur, Caitlin Black, Louise M. Emmerson, Robin Freeman, Greg Hines, Chris J. Lintott, Zuzana Macháčková, Grant Miller, Rob Simpson, Colin Southwell, Holly R. Torsey, Andrew Zisserman, Tom Hart

**Affiliations:** 1 Department of Zoology, University of Oxford, South Parks Road, Oxford OX1 3PS, UK; 2 Zooniverse, Department of Physics, University of Oxford, Denys Wilkinson Building, Keble Road, Oxford OX1 3RH, UK; 3 Department of Engineering Science, University of Oxford, Parks Road, Oxford OX1 3PJ, UK; 4 Department of the Environment and Energy, Australian Antarctic Division, 203 Channel Highway, Kingston, Tasmania 7050, Australia; 5 Institute of Zoology, Wellcome Building, Outer Circle, Regent’s Park, London NW1 4RY, UK; 6 Google UK, Belgrave House, 76 Buckingham Palace Road, London SW1W 9TQ, UK

**Keywords:** Phenology, Conservation biology, Population dynamics, Behavioural ecology

## Abstract

Automated time-lapse cameras can facilitate reliable and consistent monitoring of wild animal populations. In this report, data from 73,802 images taken by 15 different *Penguin Watch* cameras are presented, capturing the dynamics of penguin (Spheniscidae; *Pygoscelis spp.*) breeding colonies across the Antarctic Peninsula, South Shetland Islands and South Georgia (03/2012 to 01/2014). Citizen science provides a means by which large and otherwise intractable photographic data sets can be processed, and here we describe the methodology associated with the *Zooniverse* project *Penguin Watch*, and provide validation of the method. We present anonymised volunteer classifications for the 73,802 images, alongside the associated metadata (including date/time and temperature information). In addition to the benefits for ecological monitoring, such as easy detection of animal attendance patterns, this type of annotated time-lapse imagery can be employed as a training tool for machine learning algorithms to automate data extraction, and we encourage the use of this data set for computer vision development.

## Background and Summary

Camera-traps have proved an invaluable asset to numerous ecological studies, allowing large data sets to be obtained with relative ease and constant effort^1–3^. Indeed, the potential applications of camera-trap research are ever-expanding, mainly owing to significant advancements in both camera technologies and the software packages available to process their output^4,5^. When combined with other methodologies – for example niche analysis^6^ – image data sets can be used to reveal fundamental information about elusive species, such as the spatial distribution of a population^6^, abundance estimates^7^, or the nature of predation events^8^. In addition to motion-triggered camera-traps, however, it is becoming increasingly clear that there is a need for *in situ*, automated, monitoring in ecological research^9^. When cameras are used for this monitoring, they can be programmed to capture images at regular intervals (e.g. once per hour) irrespective of whether animals are present (unlike camera-traps), and thus provide a near-continuous, regular record of occupancy and observations of a population or habitat over time^10^. As with camera-traps, automated time-lapse cameras have benefitted from advances in digital technology, meaning it is now possible for them to be deployed in remote areas over long periods of time^9^. Furthermore, citizen science platforms provide a means by which large image data sets can be rapidly processed, reducing the need for time-consuming manual annotation^2^.

Due to its remote, harsh environment, large-scale on-ground monitoring studies in Antarctica are challenging and therefore rare^11^. As such, the majority of studies investigating penguin population dynamics have been focused on a specific location, or collectively several locations, with subsequent extrapolation of local data to cover a wider region or regions^11^. However, this approach is insufficient when it comes to understanding numerous wide-spread populations, especially since threats, such as over-fishing, may vary markedly between regions. Automated cameras overlooking penguin colonies were pioneered by Newbery and Southwell^12^, and a large-scale camera network was subsequently established in East Antarctica by the Australian Antarctic Division. The method has since been adopted by researchers from several nations (the United Kingdom (including the Oxford University project *Penguin Watch*), Argentina, Poland, Spain, Ukraine and the United States) working in other regions of Antarctica, and has received support from the Commission for the Conservation of Antarctic Marine Living Resources (CCAMLR). There are now over 150 cameras on penguin colonies in operation on the Antarctic continent and selected Southern Ocean islands. The construction of a collaborative remote camera network and a growing number of projects in Antarctica should address the knowledge gap of limited geographic coverage for aspects of population monitoring, and thus enhance our understanding of breeding phenology and reproductive success of penguins and other seabirds. Ultimately, a greater understanding of penguin population dynamics, reproductive success and phenology will allow the impact of threats, such as climate change and over-fishing, to be monitored and disentangled, paving the way for more effective conservation measures.

An automated camera network provides a solution to the impracticalities of year-round, large-scale manned monitoring, but the wealth of photographic information produced can be problematic for research teams to process. Citizen science can be used to dramatically increase the efficiency of data processing, thus helping to alleviate the problem of time-consuming image annotation^2^. As such, we created the website *Penguin Watch* (https://www.penguinwatch.org), a citizen science project hosted by the *Zooniverse* (https://www.zooniverse.org). Within *Penguin Watch*, volunteers are asked to classify images by tagging individuals and labelling them as ‘adult’, ‘chick’ or ‘egg’ (for penguins) or ‘other’ (which can be used to indicate the presence of other animals, humans and ships). This level of annotation is integral for down-stream analyses, as it allows detection of important phenological stages such as chick hatching. To date, over six million images have been classified by 49,520 registered volunteers and a wealth of anonymous (unregistered) participants. This report provides a detailed overview of *Penguin Watch*, from the initial collection of data via remote cameras to the production of metadata through *Zooniverse*; we also present analyses of data reliability (Tables 1, 2, 3).

### Citizen Science and Computer Vision

In addition to aiding a wide variety of research programmes, citizen science can be considered an effective strategy for public engagement and education^13^. In a survey carried out by the *Zooniverse* in 2014, 90.6% of participating volunteers stated that they “like to contribute to scientific progress,” while 84.7% of people were “fascinated by the projects” in which they were involved^14^. Individual responses also conveyed an enthusiasm for the projects and a desire to discover something new – whether it be an animal or asteroid – was a common motivation^14^.

A further benefit of citizen science is that the large quantity of information provided can be used to train machine learning algorithms to carry out the task automatically^15,16^. Computer vision techniques provide an alternative and complementary approach to photographic data processing, and there is potential to adopt both methodologies for future analyses of *Penguin Watch* data (see Arteta *et al.*, (2016)^15^ for a machine learning approach).

## Methods

### Field Methods

#### Layout of remote camera network

The *Penguin Watch* camera network currently comprises 91 units spread across the Falkland Islands, South Georgia and the South Sandwich Islands, and the Antarctic continent. Each camera generally captures images once per hour year-round (exceptions include summer ‘foraging cameras’, which take photographs once per minute), typically for a period falling between 0700 and 2000 h minimum (Table 4 (available online only)). As processing and analyses fundamentally rely on the ability to visualise penguins within images, the exact nature of the camera type, set-up and imaging frequency is flexible, with images still being comparable between studies and remaining tractable for citizen science and machine learning approaches.

#### Cameras

The images published here originate from the camera network constructed under the *Penguin Watch* program at Oxford University. All images in this data descriptor were captured by Reconyx HC500 Hyperfire trail cameras (commercially available; Reconyx Inc., Holmen, WI, USA). Each unit costs approximately $US 550, with an additional cost of $US 180 for an (optional) external rechargeable lead acid battery. The use of an external battery with a solar cell negates the need for annual servicing and internal battery changes. If powered internally, 12×AA lithium-ion batteries are required. Camera units have a lifespan of approximately three years before they become unreliable or show signs of wear.

**Figure 1:**
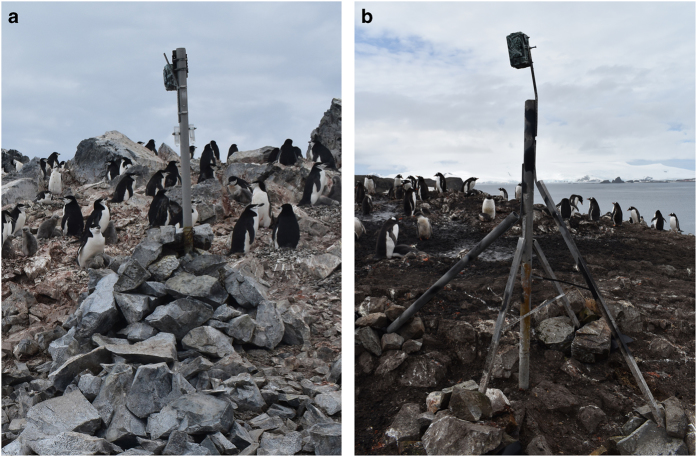
Two examples of remote camera structures. (**a**) A wire rock basket, covered by further rocks, is used to support the metal scaffold pole (Orne Harbour, Antarctic Peninsula); (**b**) multiple metal “legs” are fastened to the main structure for support; each “foot” is secured using rocks (Aitcho Islands, South Shetland Islands, Antarctic Peninsula). The design shown in (**b**) is favoured for future constructions as the “legs” provide increased stability and are longer-lasting than the wire used in (**a**), which becomes brittle after approximately three years. The cameras shown here are powered by internal batteries. Photo credit: FMJ.

Each camera is attached to a vertical metal pole, which is supported either by a wire rock basket (Fig. 1a) or by additional metal poles, which are in turn secured by rocks (Fig. 1b). In most cases, maintenance occurs once a year and involves the removal of Secure Digital (SD) cards and the insertion of new batteries. With the addition of large external power supplies, cameras could be left in place for multiple years and, if necessary, the frequency at which images are captured may also be altered to monitor fine-scale behaviour.

The use of time-lapse cameras as opposed to camera-traps fitted with passive infrared (PIR) sensors is advantageous for a number of reasons. Firstly, the PIR detector may be insufficient to pick up heat signals from distant penguin bodies, while images from time-lapse cameras can show penguins in the far distance. These individuals may or may not belong to the colony being monitored; regardless, knowledge of their presence is useful ecological information. Furthermore, it is important to obtain data from constant time intervals to perform analyses (such as mark-recapture studies) on the data. Finally, it would be difficult to ensure that a camera-trap has enough power to continue operating throughout an entire year using a PIR sensor. Increased function in the summer months could lead to camera failure before battery replacement, meaning the colony could go unmonitored during crucial stages of their annual cycle (e.g. the arrival of adults in the spring).

### Data and Analytical Methods

Analysis-ready images (i.e. renamed and resized raw files) were prepared using loops in R^17^. An Exchangeable Image File (EXIF) tool (https://sourceforge.net/p/exiftool/code/ci/master/tree/) was used to extract metadata, such as date/time information and temperature, from each photograph. The image processing script can be found on GitHub and Figshare (see ‘Code Availability’). It should be noted that the images published alongside this Data Descriptor (Table 4 (available online only); Data Citation 1) have already been renamed, but have not been resized – they retain their original dimensions of either 1920×1080 pixels or 2048×1536 pixels. During the resizing process, aspect ratios are maintained: 1920×1080 pixel images are resized to 1000×562.5 pixels (aspect ratio=0.5625), and 2048×1536 pixel images are resized to 1000×750 pixels (aspect ratio=0.75). See Data Citation 1 for the raw images and associated metadata.

#### Image Annotation

**Figure 2:**
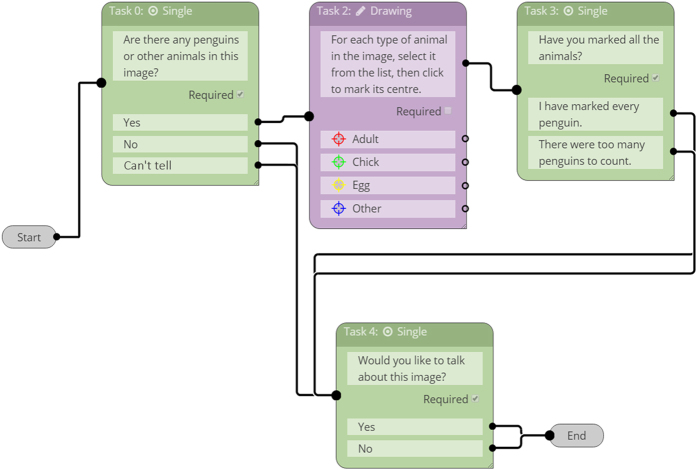
The *Penguin Watch* volunteer work flow. If animals are present in a given image, volunteers are asked to tag individuals by clicking on them, and classify them as ‘adult’, ‘chick’, ‘egg’, or ‘other’ (the latter can be used to identify other fauna, ships or humans). Once an image has been classified, volunteers are given the opportunity to ‘talk’ about it on a *Penguin Watch* forum. Green boxes indicate that volunteers must supply an answer, purple boxes indicate that a process must be carried out (such as clicking on penguins). Image source: https://www.zooniverse.org/lab.

The *Penguin Watch* citizen science project (*v 1.0* launched September 17^th^ 2014) is the product of a collaboration with the citizen science platform *Zooniverse* (https://www.zooniverse.org). *Penguin Watch* operates on the Ouroboros Application Programming Interface (API), is scripted in Ruby on Rails, and asks volunteers to classify images through a JavaScript interface (via a regular web browser). Citizen scientists are presented with a single image, and follow the workflow outlined in Fig. 2.

As with all *Zooniverse* projects, each subject is viewed by multiple volunteers in order to increase data reliability. Specifically, if *any* volunteer identifies animals in an image, that image is shown to ten people by default. If the first four volunteers state that no animals are present, or they are unsure (indicated by an ‘I can’t tell’ option), that image is then retired (assuming that nobody has identified any animals), meaning it is removed from the active data set and not seen by any further volunteers. This practice of retiring blank images is also employed by the *Snapshot Serengeti* citizen science project^2,18^. Classification data are stored in MongoDB, a NoSQL database which records data in a BSON (Binary JavaScript Object Notation) format^2^. Volunteer information is listed for each annotation, as either an anonymised IP address hash (non-logged in volunteers) or a unique *Zooniverse* identification number (logged in volunteers). Source code for the *Penguin Watch* project can be accessed online (see ‘Code Availability’).

#### Clustering Algorithm

**Figure 3:**
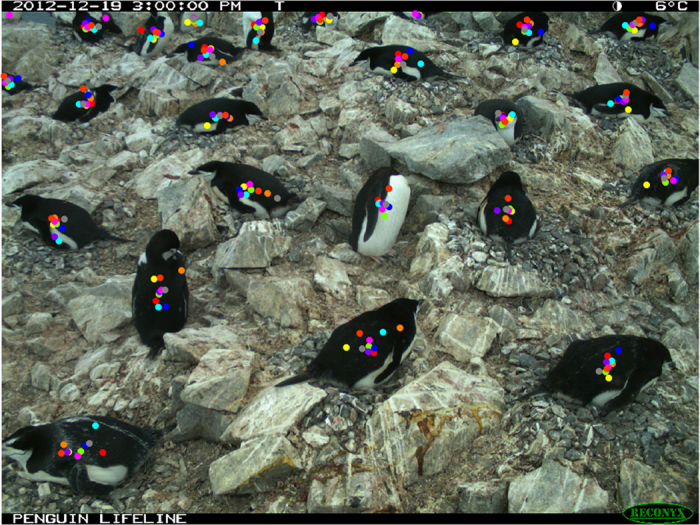
Image HALFb2013a_000051.JPG, with the ‘raw clicks’ of *Penguin Watch* volunteers overlaid. Each dot represents a single click, with colours specific to ten individual volunteers (images said to contain animals are shown to ten volunteers by default). Using the clustering algorithm (see ‘Code Availability’), ‘consensus clicks’ are derived from each group of markings. The coordinates of ‘raw clicks’ and ‘consensus clicks’ can be found in the Dryad Digital Repository (Tables 5 and 6; Data Citation 1).

Since each *Penguin Watch* image is classified by multiple citizen scientists, a clustering algorithm is required to generate ‘consensus click’ data for each annotated object (penguin or ‘other’). In other words, the coordinates of clicks made on an image (by multiple volunteers – see Fig. 3 for an example) are grouped by their spatial position, so that an average xy coordinate is produced for each tightly clustered group of markings.

This is achieved through agglomerative hierarchical clustering, using Ward’s method to cluster based on the Euclidean distance between points. Agglomerative hierarchical clustering allows the user to choose the level of final clustering, and therefore the total number of clusters, using relevant data properties. Visually, this is represented by a dendrogram showing the number of clusters at each agglomerative step, with the ‘leaves’ of the tree being single-marking clusters, and the ‘root’ a cluster containing all markings. The final clusters for each image were chosen by traversing each branch of the dendrogram tree and selecting a level for that branch which maximized the number of clicks per cluster while also satisfying the requirement that no user clicked twice within the same cluster. This choice is founded in the logic that, while different volunteers’ clicks on a given penguin may be shifted slightly from one another, each volunteer will click an individual penguin only once, meaning that two separate clicks made by one person represent two penguins even if those clicks are near each other. When every marking has been assigned to a cluster, an average xy coordinate (using the median average, which is more robust to outliers than the mean average) – or ‘consensus click’ – can be calculated for each grouping. The aggregation script (written in Python v2.7) is publicly available (see ‘Code Availability’).

Output data (see Table 5) are exported in comma-separated values (csv) file format and comprise of: 1) image name, 2) ‘consensus click’ coordinates, 3) estimated probability (P) values for adults, chicks and eggs, 4) the estimated probability (P) of a true positive, and 5) the number of markings that contributed to each classified individual (Data Citation 1). The probability value for an adult/chick/egg refers to the estimated probability that the corresponding individual is indeed an adult/chick/egg, based on the number of volunteers assigning it this label, as a proportion of the total number of clicks on that individual. Similarly, the probability of a true positive is the estimated probability that an individual is actually present at the location of the ‘consensus click’, given as the proportion of volunteers marking the area out of the total number of volunteers who indicated that animals were present in the image. Therefore, the number of volunteers who selected ‘yes’ when asked ‘Are there any penguins or other animals in this image?’ can be quickly calculated by dividing the number of markings by the probability of a true positive. The 'number of markings' is simply the number of clicks that contributed to the 'consensus click'. This allows the researcher to decide on a probability threshold for subsequent analysis (e.g. P>0.5 is treated as a true positive), and to filter out classifications with less than a set number of markings. For example, if a cluster was formed from only one click, it is likely that it was made in error, and should not be carried forward.

Sometimes it is necessary to filter the raw clicks prior to performing the cluster analysis. For example, *Penguin Watch* volunteers are very occasionally shown the same image more than once. Following the logic of the aggregation algorithm (where clicks made by the same volunteer are placed in separate clusters), if duplicate markings are not removed, multiple clusters will be erroneously created, leading to an overestimate of penguin numbers. In this data set, 0.28% of raw clicks were not incorporated into consensus clicks, either owing to this duplication, or cluster failure (in rare cases clicks can be missed by the aggregation algorithm). Furthermore, when using a mobile phone to operate *Penguin Watch*, volunteers can sometimes drag the marker tool beyond the boundaries of an image. A click made in this area will be carried forward, but the coordinate values will lie beyond the limit of the image (i.e. x<0 or >1000, y<0 or >562.5 or >700) and can therefore be easily identified. These accidental markings can be filtered out prior to clustering, or – as is the case in the accompanying data set (Data Citation 1) – can be removed during subsequent analysis (~97% of these markings are filtered out using the lowest threshold level of ‘>1 marking’ required to form a consensus click).

### Code Availability

All data sets are processed in *R* (currently v3.4.1) to produce analysis-ready image files. The ‘rename and resize’ script can be accessed via GitHub at https://github.com/zooniverse/Data-digging/blob/master/example_scripts/Penguin_Watch/Penguin_Watch_ImageProcessingScript.R, and a static version is archived on Figshare^19^. The aggregation script (clustering algorithm) is written in Python (v2.7) and can be found at https://github.com/zooniverse/aggregation/blob/master/penguins/aggregate.py. A static version of this script is also archived on Figshare^20^. Source code for *Penguin Watch* is located at https://github.com/zooniverse/penguinwatch.

## Data Records

**Figure 4:**
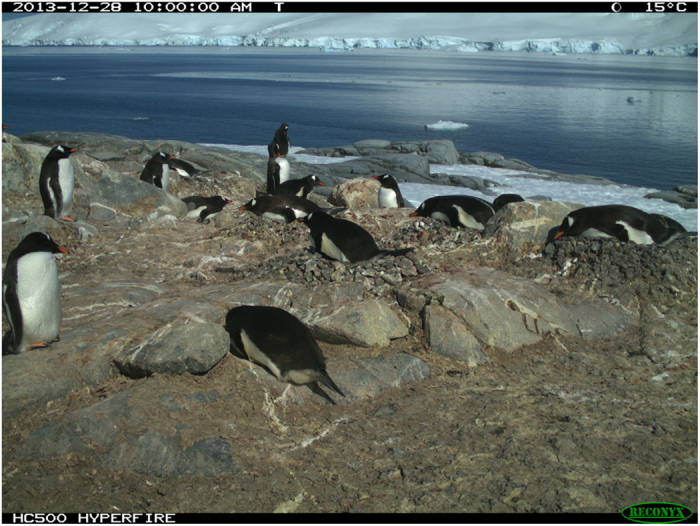
A sample image (DAMOa2014a_000028.JPG), taken by a Reconyx time-lapse camera at Damoy Point, Weincke Island, Antarctic Peninsula (64.82° S, 63.49° W). Date, time, moon phase and temperature information is shown at the top of the image. This is one of 73,802 photographs that can be found online in the Dryad Digital Repository (Data Citation 1).

A total of 73,802 raw image files retrieved from 15 *Penguin Watch* time-lapse cameras, collected between March 2012 and January 2014, are stored in the Dryad Digital Repository (Table 4 (available online only); Data Citation 1). An example image can be seen in Fig. 4. The images are in JPEG format and range in number from 137 to 10,732 files per camera for a single year (Table 4 (available online only)). This variation in quantity exists owing to differing operational durations and frequencies of image capture: for example, MAIVb2013c (137 photographs) contains hourly footage (0800–2000) from 11 days, whereas PETEc2014 (10,732 photographs) comprises images taken every half hour (0700-2130) from January 2013 to January 2014. While differing in duration, each image set is complete; i.e. no photographs have been discarded and there are no irregular temporal gaps in the data sets. Every image is uploaded to *Penguin Watch* – if visibility is poor (owing to low light levels or an obscured lens for example), volunteers can respond with ‘I can’t tell’ when asked if any animals are present. *Penguin Watch* ‘consensus click’ data and metadata associated with each of the 73,802 images can also be found in the Dryad Digital Repository (Tables 5 and 6; Data Citation 1). Raw images are grouped into 23 separate folders based on camera and year. There are 34 ‘consensus click’ folders and 34 corresponding metadata folders, as some camera/year combinations have been subdivided.

### Explanation of terms

#### ‘Consensus click data’

‘Consensus click data’ are the data produced via the clustering algorithm discussed in ‘Methods’. Each row contains the following information:

*Name*: Unique image reference for identification, in the format: SITExYEARx_imagenumber.csv; e.g. DAMOa2014a_000001.csv.

*x_centre*: x coordinate value (in pixels) for the ‘consensus click’ (i.e. the coordinate calculated by the clustering algorithm). The origin (point 0, 0) is located in the top left-hand corner of the image, meaning it may be necessary to reverse the y-axis of a plot in order to overlay the consensus clicks correctly. One coordinate value denotes one individual penguin/’other’.

*y_centre*: y coordinate value (in pixels) for the ‘consensus click’ (i.e. the coordinate calculated by the clustering algorithm). The origin (point 0, 0) is located in the top left-hand corner of the image, meaning it may be necessary to reverse the y-axis of a plot in order to overlay the consensus clicks correctly. One coordinate value denotes one individual penguin/’other’.

*probability_of_adult*: Estimated probability that the corresponding individual is an adult – based on the number of volunteers classifying it as such, as a proportion of the total number of clicks on that individual.

*probability_of_chick*: Estimated probability that the corresponding individual is a chick – based on the number of volunteers classifying it as such, as a proportion of the total number of clicks on that individual.

*probability_of_egg*: Estimated probability that the corresponding marking indicates an egg – based on the number of volunteers classifying it as such, as a proportion of the total number of clicks on that area.

*probability_of_true_positive*: Estimated probability of an individual (of any ‘type’; adult, chick, egg or other) being present at the location of the coordinates. This is the proportion of volunteers marking the penguin out of the total number volunteers who click ‘yes’ when asked ‘Are there any penguins or other animals in this image?’.

*num_markings*: The number of volunteer clicks that were aggregated to produce the ‘consensus click’ coordinate values (i.e. the number of individual clicks on a specific area of the image).

#### Anonymised raw classifications and metadata

These data include the coordinates for raw classifications, which provide the input data for the aggregation script (see ‘Methods’). Environmental metadata, such as temperature, are also provided.

*user_name (anonymised)*: *Zooniverse* unique volunteer name (or IP address), here anonymised.

*subject_zooniverse_id*: Unique identification code assigned to each image within *Zooniverse*.

*lunar_phase*: Moon phase when the image was captured (one of eight options: “full” (full), “new” (new), “newcres” (new crescent), “firstq” (first quarter), “waxinggib” (waxing gibbous), “waninggib” (waning gibbous), “lastq” (last quarter) or “oldcres” (old crescent)).

*original_size_x*: Image dimension (x axis) prior to resizing for *Zooniverse*; either 1920 or 2048 pixels (note: aspect ratios are maintained during resizing).

*original_size_y*: Image dimension (y axis) prior to resizing for *Zooniverse*; either 1080 or 1536 pixels (note: aspect ratios are maintained during resizing).

*path*: Folder pathway, which includes the image name (e.g. DAMOa/DAMOa2014a_000025).

*temperature_f*: Temperature (in degrees Fahrenheit, as recorded by the camera) at the time the photograph was taken.

*timestamp*: Time and date information for the image, in the format YYYY:MM:DD HH:MM:SS.

*animals_present*: Indication by volunteers that animals are present in a given image. In the format ‘yes’ (present), ‘no’ (absent) or ‘can’t tell’.

*all_marked*: Whether volunteers think that they have marked all the individuals (including all adults, chicks, eggs and others) in an image. In the format ‘complete’ or ‘incomplete’.

*value*: Volunteer classification for the ‘type’ of individual: ‘adult,’ ‘chick,’ ‘egg,’ or ‘other.’

*x*: x coordinate value (in pixels) for an individual click (note, not a ‘consensus click’ – see ‘*x_centre*’). The origin (point 0, 0) is located in the top, left-hand corner of the image.

*y*: y coordinate value (in pixels) for an individual click (note, not a ‘consensus click’ – see ‘*y_centre*’). The origin (point 0, 0) is located in the top, left-hand corner of the image.

## Technical Validation

To examine the agreement between aggregated volunteer clicks and ‘expert’ annotations – and thus the reliability of *Penguin Watch* as a data processing tool – classifications made by authors CB and FMJ were employed as a ‘gold standard’ (GS). It should be noted that all processes (GS, citizen science (CS), and computer vision) have false positives and negatives, which we can only estimate without knowledge of the underlying truth. Analysis of the data for 100 images (spanning those published here, and the wider *Penguin Watch* database), randomly selected from the GS (CB) classifications that stated no animals were present, revealed 96% agreement between the GS and aggregated volunteer clicks (i.e. 96% were marked as containing no animals). Of the remaining 4%, three images had been erroneously marked by volunteers, and one image contained animals which had been missed in the GS (albeit there were also six erroneous volunteer classifications in this image). In all cases where there were false positives, only one volunteer had marked the image. Thus, if a minimum threshold for *num_markings* were employed (> 1 marking or above), each of these would be filtered out during subsequent analyses.

Image classifications from four cameras – DAMOa, HALFc, LOCKb, and PETEc – were also analysed. These cameras were selected as they represent a range of ‘views’ (i.e. the cameras are positioned at different angles and distances from a colony), and monitor each of the three *Pygoscelis* species – *Pygoscelis papua* (Gentoo; DAMOa, LOCKb), *P. antarcticus* (Chinstrap; HALFc), and *P. adeliae* (Adélie; PETEc). GS (FMJ) classifications fulfilling two criteria – 1) the image contained animals, and 2) images were marked as complete – were selected. A random sample of 300 images was obtained for each camera, with the exception of HALFc, where the whole sample of 283 photographs was used (see Table 4 (available online only)).

Number of adults, number of chicks, and number of adults and chicks combined, as calculated using *Penguin Watch* data, were compared to the GS counts. Probability thresholds for adults and chicks were set to P>0.5 for this analysis (see ‘Explanation of terms’). Four different threshold levels for *num_markings* were applied to the *Penguin Watch* classifications. For example, a threshold level of greater than two (>2) requires three or more *Penguin Watch* participants to click on a ‘penguin’ for it to be counted. Average differences, measured in number of penguins, between the GS count and the count produced using each threshold level, are shown in Tables 1 and 2. The proportion of differences that were either zero (i.e. the GS and *Penguin Watch* data showed the same result) or one are also shown for each category.

The results show that accuracy – where higher accuracy is defined as a smaller average difference between the GS and CS data – varies depending on site, threshold level, and whether adults and chicks are considered together or separately. However, the most accurate results for adults at all four sites were obtained using a threshold level of >3, where the average differences ranged from 0.88 (σ=1.34) to 2.36 (σ=2.69) penguins (Table 1). At this threshold level, the number of false positives and false negatives is at a minimum. If the threshold level is increased further, the proportion of overestimated values continues to decrease (as more clicks are required for an ‘individual’ to be counted), but the proportion of underestimated values increases (Table 3). For the sites examined here, a threshold of >3 reflects an appropriate balance between over- and under-estimation. Since the four sites vary in camera angle and species, this threshold level would be a suitable starting point when analysing data from different cameras.

The greatest deviation from the GS (with an average difference of 2.36 adults) is associated with PETEc. This camera captured the greatest number of individuals on average (36.43 compared to 11.79, 15.03 and 10.99 at DAMOa, HALFc and LOCKb, respectively). Greater error with increasing colony size is expected, and suggests that citizen scientists have more difficulty distinguishing individuals when presented with larger groups. Segmentation of the image to focus on a region of interest may offer a partial solution to this problem, although obscuration of individuals by their conspecifics remains an issue.

The accuracy of chick counts (Table 2) – where lowest average differences range from 0.25 (σ=0.68) to 1.89 (σ=2.69), and the proportion of differences equating to zero or one is as high as 94% for DAMOa (>1) – appears to be greater than that of the adult counts. However, this is owing to the large number of images that do not contain chicks (according to the GS and CS data). If these images are removed, the accuracy decreases, with the lowest values at each site ranging from 1.18 (σ=1.09) to 3.17 (σ=2.85).

**Figure 5:**
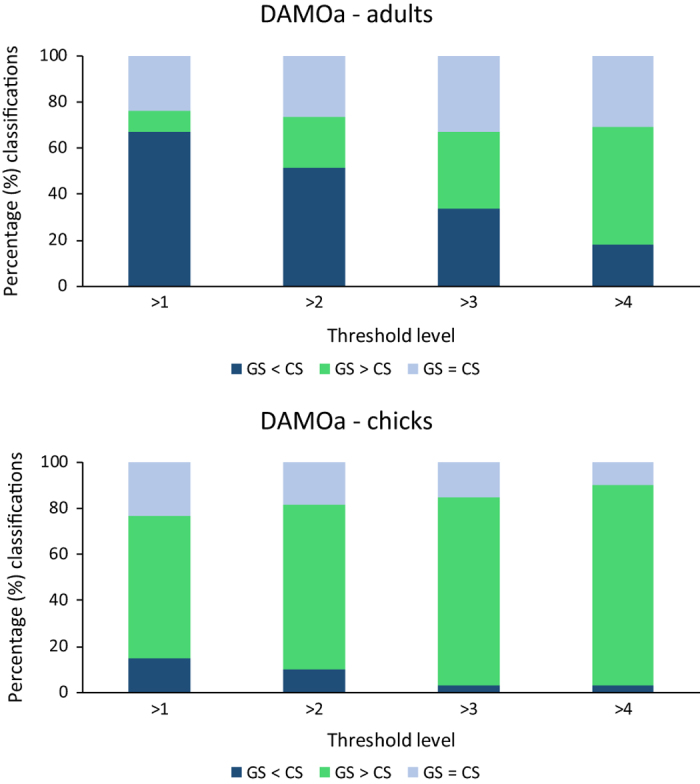
Percentage of *Penguin Watch* (CS) classifications that are greater than (GS < CS), less than (GS > CS) or equal to (GS = CS) gold standard (GS) classifications (i.e. overestimates, underestimates or matches) for DAMOa. Top: Adult classifications only (n=300); bottom: chick classifications only (for images where chicks are present according to GS and/or CS data; n=60).

One explanation for the lower accuracy of chick estimation compared to adults could be misinterpretation of individuals (i.e. labelling a chick as an adult, or vice versa). This could occur when chicks are losing their down feathers, and thus look similar to adult birds. However, if this was the main issue, accuracy levels should be optimised when adult and chick counts are combined, which is not the case (Table 1). Instead, the data suggest that chicks are often missed by volunteers. While overestimation is more common than underestimation at the >1 and >2 threshold level in adults, the proportion of underestimated values consistently outweighs the proportion of overestimates at each threshold level for chick counts, as shown in Fig. 5 and Table 3.

Chicks are often partially concealed by a parent, particularly during the brood-guard stage, which may explain why they are sometimes missed. Increased training on the *Penguin Watch* interface – for example through the addition of imagery showing very young chicks, and species-specific information on expected lay dates – may help to overcome this issue. It should also be noted that different classification thresholds may be appropriate for adults and chicks. For example, chick accuracy was optimised at the >1 level at the DAMOa, HALFc and PETEc sites. Where chicks are often missed, it is logical that fewer volunteers should be required to tag them in order for them to be recognised.

A second reason for the underestimation of chicks could stem from the *Penguin Watch* work-flow. After a volunteer has classified 30 individuals, they are given the option of moving on to the next image, to maintain interest. The results suggest that this is not detrimental to the accuracy of adult counts, with the combined efforts of each volunteer covering an entire image (counts were overestimated in 79.33% of cases at PETEc (>1 threshold), where the number of individuals frequently exceeds 30). However if the majority of volunteers choose to tag all the adults first, before classifying any chicks, chicks may be consistently missed in images containing >30 penguins. An alteration to the *Penguin Watch* tutorial, requesting that volunteers classify a variety of penguin ‘types’ before moving onto a new image, may help to overcome this.

Through careful selection of threshold levels, consideration of associated error, and continued evaluation of data quality, *Penguin Watch* can provide us with a reliable means with which to process large quantities of imagery, which would otherwise be intractable.

## Additional information

**How to cite this article**: Jones, F. M. *et al.* Time-lapse imagery and volunteer classifications from the Zooniverse Penguin Watch project. *Sci. Data* 5:180124 doi: 10.1038/sdata.2018.124 (2018).

**Publisher**’**s note**: Springer Nature remains neutral with regard to jurisdictional claims in published maps and institutional affiliations.

## Supplementary Material



## Figures and Tables

**Table 1:** Comparison of counts between *Penguin Watch* (CS) and GS data for four cameras: DAMOa, HALFc, LOCKb and PETEc. ‘Threshold employed’ relates to the filtering threshold applied during analysis.

	Threshold employed							
	Only **adult** classifications				All **adult** and **chick** classifications			
	>1	>2	>3	>4	>1	>2	>3	>4
**DAMOa**	n=300				n=300			
Average difference	1.88	1.50	1.18	1.24	1.94	1.58	1.37	1.53
σ	2.15	1.78	1.56	1.40	2.22	1.86	1.69	1.61
Proportion of differences that are 0 or 1	0.56	0.67	0.75	0.69	0.56	0.64	0.69	0.62
**HALFc**	n=283				n=283			
Average difference	1.28	0.99	0.88	0.99	1.45	1.27	1.34	1.55
σ	2.06	1.58	1.34	1.28	2.08	1.71	1.64	1.72
Proportion of differences that are 0 or 1	0.74	0.82	0.85	0.82	0.69	0.71	0.68	0.64
**LOCKb**	n=300				n=300			
Average difference	1.41	1.25	1.17	1.19	1.40	1.24	1.18	1.29
σ	2.31	2.12	1.98	1.96	2.30	1.89	1.74	1.78
Proportion of differences that are 0 or 1	0.73	0.77	0.77	0.75	0.72	0.73	0.73	0.68
**PETEc**	n=300				n=300			
Average difference	3.60	2.48	2.36	3.27	4.29	3.54	4.37	6.06
σ	3.50	2.99	2.69	2.86	4.51	4.07	4.41	5.20
Proportion of differences that are 0 or 1	0.28	0.46	0.46	0.30	0.25	0.37	0.33	0.20
For example, a threshold of ‘>2’ means that at least three people must have marked an area before it is counted as a penguin. ‘Average difference’ is the mean average of all differences between the GS and *Penguin Watch* counts, measured in number of individuals. ‘Proportion of differences that are 0 or 1’ is the proportion of images for which the *Penguin Watch* count was either equal to the GS, or different by one individual (either higher or lower), respectively. The results for adults, and adults and chicks combined, are presented here. To be counted as an adult or a chick, *probability_of_adult* or *probability_of_chick* was >0.5 (see ‘Explanation of terms’).								

**Table 2:** Comparison of chick counts between *Penguin Watch* (CS) and GS data for four cameras: DAMOa, HALFc, LOCKb and PETEc; see Table 1 legend for definitions.

	Threshold employed							
	Only **chick** classifications (all images)				Only **chick** classifications			
	>1	>2	>3	>4	>1	>2	>3	>4
**DAMOa**	n=300				n=60			
Average difference	0.25	0.27	0.29	0.33	1.25	1.33	1.45	1.65
σ	0.68	0.71	0.75	0.85	1.04	1.05	1.06	1.20
Proportion of differences that are 0 or 1	0.94	0.93	0.92	0.91	0.68	0.67	0.60	0.53
**HALFc**	n=283				n=109			
Average difference	0.46	0.51	0.58	0.65	1.18	1.31	1.50	1.69
σ	0.89	0.99	1.11	1.24	1.09	1.22	1.35	1.50
Proportion of differences that are 0 or 1	0.90	0.87	0.83	0.82	0.73	0.65	0.57	0.54
**LOCKb**	n=300				n=122			
Average difference	0.94	0.90	0.88	0.92	2.32	2.22	2.16	2.25
σ	2.21	2.08	2.07	2.12	2.97	2.79	2.79	2.84
Proportion of differences that are 0 or 1	0.81	0.81	0.83	0.80	0.53	0.53	0.59	0.52
**PETEc**	n=300				n=179			
Average difference	1.89	2.07	2.49	3.06	3.17	3.46	4.17	5.12
σ	2.69	2.69	3.12	3.70	2.85	2.71	3.06	3.51
Proportion of differences that are 0 or 1	0.60	0.55	0.52	0.49	0.33	0.25	0.20	0.15
The first column shows the results for all images in the sample, including those where – according to the GS and CS data – no chicks were present. The second column presents the results for the sample of images where chicks were present, according to the GS and/or CS classifications. When extracting *Penguin Watch* data, *probability_of_chick* was set to >0.5 (see ‘Explanation of terms’).								

**Table 3:** Percentage of *Penguin Watch* (CS) classifications that are greater than (GS < CS), less than (GS > CS) or equal to (GS = CS) gold standard (GS) classifications (i.e. overestimates, underestimates or matches) for DAMOa, HALFc, LOCKb and PETEc.

	Threshold employed							
	Only **adult** classifications				Only **chick** classifications			
	>1	>2	>3	>4	>1	>2	>3	>4
**DAMOa**	n=300				n=60			
GS<CS (%)	67.00	51.67	33.67	18.00	15.00	10.00	3.33	3.33
GS>CS (%)	9.33	22.00	33.33	51.33	61.67	71.67	81.67	86.67
GS=CS (%)	23.67	22.33	33.00	30.67	23.33	18.33	15.00	10.00
**HALFc**	n=283				n=109			
GS<CS (%)	48.06	35.34	12.61	14.49	17.43	11.01	2.75	0.92
GS>CS (%)	12.72	20.14	32.16	48.76	57.80	63.30	73.39	79.82
GS=CS (%)	39.22	44.52	45.23	36.75	24.78	25.69	23.85	19.27
**LOCKb**	n=300				n=122			
GS<CS (%)	48.67	37.67	27.33	22.33	27.05	23.77	17.21	9.84
GS>CS (%)	11.00	18.33	26.67	32.00	51.64	54.92	63.11	66.39
GS=CS (%)	40.33	44.00	46.00	45.67	21.31	21.31	19.67	23.77
**PETEc**	n=300				n=179			
GS<CS (%)	79.33	55.33	26.33	13.67	32.96	20.67	10.06	6.70
GS>CS (%)	13.00	30.67	54.00	77.00	58.66	72.07	83.24	91.60
GS=CS (%)	7.67	14	19.67	9.33	8.38	7.26	6.70	1.68
Results are shown for adult classifications (all images) and chick classifications (only images where chicks are present, according to GS and/or CS data).								

**Table 4:** Raw Images – Metadata and File Information.

Site (camera location)	Geographic coordinates	Camera name	Image set (name in repository)	File information and camera settings
Damoy Point, Weincke Island, Antarctic Peninsula	64.82° S, 63.49° W	DAMOa	DAMOa2014a	No. of files: 407Time range: 26/12/2013 1300–22/01/2014 1400Settings: 0700–2100; hourly
George’s Point, Rongé Island on the Errera Channel, Antarctic Peninsula	64.67° S, 62.67° W	GEORa	GEORa2013	No. of files: 4403Time range: 22/12/2012 1000–24/12/2013 0800Settings: 0800–1900; hourly
Half Moon Island, South Shetland Islands	62.60° S, 59.90° W	HALFb	HALFb2013a	No. of files: 306Time range: 15/12/2012 1700–08/01/2013 1000Settings: 0700–1900; hourly
		HALFc	HALFc2013a	No. of files: 283Time range: 21/12/2012 1700–09/01/2013 1400Settings: 0700–2100; hourly
Port Lockroy, Wiencke Island, Antarctic Peninsula	64.82° S, 63.49° W	LOCKb	LOCKb2013	No. of files: 2372Time range: 13/12/2012 1100–31/05/2013 1600Settings: 0700–2000; hourly
Maiviken, South Georgia	54.24° S, 36.50° W	MAIVb	MAIVb2012a	No. of files: 660Time range: 13/10/2012 0900–03/01/2013 1300Settings: 0900–1100, 1300–1700; hourly
			MAIVb2013a	No. of files: 3878Time range: 03/01/2013 1400–28/10/2013 1700Settings: 0800–2000; hourly
			MAIVb2013c	No. of files: 137Time range: 29/10/2013 0800–08/11/2013 1400Settings: 0800–2000; hourly
		MAIVc	MAIVc2013	No. of files: 4661Time range: 15/10/2012 0900–08/11/2013 1300Settings: 0900–1100, 1300–1700; hourly
Neko Harbour, Andvord Bay, Antarctic Peninsula	64.86° S, 62.52° W	NEKOa	NEKOa2012a	No. of files: 1608Time range: 03/03/2012 1000–25/11/2012 1500Settings: 1000–1500; hourly
			NEKOa2013	No. of files: 3552Time range: 26/11/2012 1000–25/12/2013 1500Settings: 0800–1600; hourly
			NEKOa2014a	No. of files: 239Time range: 25/12/2013 1600–13/01/2014 0700Settings: 0700–1900; hourly
		NEKOb	NEKOb2013	No. of files: 6449Time range: 02/03/2012 1600–26/11/2012 0800Settings: 0000–1200; hourly
		NEKOc	NEKOc2013	No. of files: 4415Time range: 14/12/2012 1600–19/11/2013 1000Settings: 0700–1900; hourly
			NEKOc2014b	No. of files: 296Time range: 25/12/2013 1600–13/01/2014 0700Settings: 0600-2100; hourly
Petermann Island, Antarctic Peninsula	65.17° S, 64.14° W	PETEc	PETEc2013	No. of files: 506Time range: 03/12/2012 1600–11/01/2013 1400Settings: 0800–1100, 1300–2100; hourly
			PETEc2014	No. of files: 10,732Time range: 11/01/2013 1430–04/01/2014 1000Settings: 0700–2130; half hourly
		PETEd	PETEd2013	No. of files: 10,732Time range: 11/01/2013 1430–04/01/2014 1000Settings: 0700–2130; half hourly
		PETEe	PETEe2013	No. of files: 3874Time range: 12/12/2012 1600–04/01/2014 0900Settings: 0900–1800; hourly
		PETEf	PETEf2014a	No. of files: 8271Time range: 24/12/2012 1300–04/01/2014 1000Settings: 0100–1100, 1300–2300; hourly
Spigot Peak, Orne Harbour, Antarctic Peninsula	64.63° S, 62.57° W	SPIGa	SPIGa2012a	No. of files: 390Time range: 25/11/2012 1700–21/12/2012 1600Settings: 0700 -2100; hourly
			SPIGa2013b	No. of files: 5221Time range: 09/01/2013 1500–23/12/2013 1500Settings: 0700–2100; hourly
			SPIGa2014	No. of files: 410Time range: 23/12/2013 1600–21/01/2014 2000Settings: 0700–2000; hourly
	Total no. of files: 73,802			
Metadata and file information associated with each of the 73,802 ‘Raw images’ included in the Dryad Digital Repository (Data Citation 1). These images are stored under the folders: DAMO to MAIV, NEKO, PETE.1, PETE.2 and SPIG.Images retain their original dimensions (either 1920×1080 pixels or 2048×1536 pixels), but have been renamed. The *R* script used to rename files can be found on GitHub at https://github.com/zooniverse/Data-digging/blob/master/example_scripts/Penguin_Watch/Penguin_Watch_ImageProcessingScript.R and on Figshare^19^ (static version).				

**Table 5:** *Penguin Watch* Consensus Click Data.

Camera name	Corresponding image set (see Table 4)	File name (in repository)
DAMOa	DAMOa2014a	DAMOa2014a_concl.csv
GEORa	GEORa2013	GEORa2013a_concl.csv
		GEORa2013b_concl.csv
HALFb	HALFb2013a	HALFb2013a_concl.csv
HALFc	HALFc2013a	HALFc2013a_concl.csv
LOCKb	LOCKb2013	LOCKb2013a_concl.csv
		LOCKb2013b_concl.csv
MAIVb	MAIVb2012a	MAIVb2012a_concl.csv
	MAIVb2013a	MAIVb2013a_concl.csv
	MAIVb2013c	MAIVb2013c_concl.csv
MAIVc	MAIVc2013	MAIVc2013_concl.csv
NEKOa	NEKOa2012a	NEKOa2012a_concl.csv
	NEKOa2013	NEKOa2013a_concl.csv
		NEKOa2013b_concl.csv
		NEKOa2013c_concl.csv
	NEKOa2014a	NEKOa2014a_concl.csv
NEKOb	NEKOb2013	NEKOb2013_concl.csv
NEKOc	NEKOc2013	NEKOc2013a_concl.csv
		NEKOc2013b_concl.csv
		NEKOc2013c_concl.csv
	NEKOc2014b	NEKOc2014b_concl.csv
PETEc	PETEc2013	PETEc2013a_concl.csv
		PETEc2013b_concl.csv
	PETEc2014	PETEc2014a_concl.csv
		PETEc2014b_concl.csv
PETEd	PETEd2013	PETEd2013a_concl.csv
		PETEd2013b_concl.csv
PETEe	PETEe2013	PETEe2013a_concl.csv
		PETEe2013b_concl.csv
PETEf	PETEf2014a	PETEf2014a_concl.csv
SPIGa	SPIGa2012a	SPIGa2012a_concl.csv
	SPIGa2013b	SPIGa2013b_concl.csv
	SPIGa2014	SPIGa2014a_concl.csv
		SPIGa2014b_concl.csv
List of ‘PW Anonymised Raw Classifications and Metadata’ files stored in the Dryad Digital Repository (Data Citation 1), and their associated cameras/image files. The number of unique *Penguin Watch* volunteers that contributed classifications to each file are also shown. Metadata are extracted using *R* code found on GitHub at https://github.com/zooniverse/Data-digging/blob/master/example_scripts/Penguin_Watch/Penguin_Watch_ImageProcessingScript.R and Figshare^19^ (static version).		

**Table 6:** *Penguin Watch* Anonymised Raw Classifications and Metadata.

Camera name	Corresponding image set (see Table 4)	File name (in repository)	Number of unique annotators
DAMOa	DAMOa2014a	DAMOa2014a_metadata.csv	3042
GEORa	GEORa2013	GEORa2013a_metadata.csv	1810
		GEORa2013b_metadata.csv	13,353
HALFb	HALFb2013a	HALFb2013a_metadata.csv	2433
HALFc	HALFc2013a	HALFc2013a_metadata.csv	2211
LOCKb	LOCKb2013	LOCKb2013a_metadata.csv	1286
		LOCKb2013b_metadata.csv	8959
MAIVb	MAIVb2012a	MAIVb2012a_metadata.csv	3977
	MAIVb2013a	MAIVb2013a_metadata.csv	11,367
	MAIVb2013c	MAIVb2013c_metadata.csv	1177
MAIVc	MAIVc2013	MAIVc2013_metadata.csv	13,543
NEKOa	NEKOa2012a	NEKOa2012a_metadata.csv	6874
	NEKOa2013	NEKOa2013a_metadata.csv	705
		NEKOa2013b_metadata.csv	11,068
		NEKOa2013c_metadata.csv	2411
	NEKOa2014a	NEKOa2014a_metadata.csv	1831
NEKOb	NEKOb2013	NEKOb2013_metadata.csv	15,789
NEKOc	NEKOc2013	NEKOc2013a_metadata.csv	28
		NEKOc2013b_metadata.csv	1019
		NEKOc2013c_metadata.csv	13,523
	NEKOc2014b	NEKOc2014b_metadata.csv	2212
PETEc	PETEc2013	PETEc2013a_metadata.csv	2033
		PETEc2013b_metadata.csv	1926
	PETEc2014	PETEc2014a_metadata.csv	21,009
		PETEc2014b_metadata.csv	4492
PETEd	PETEd2013	PETEd2013a_metadata.csv	21,280
		PETEd2013b_metadata.csv	4594
PETEe	PETEe2013	PETEe2013a_metadata.csv	981
		PETEe2013b_metadata.csv	12,469
PETEf	PETEf2014a	PETEf2014a_metadata.csv	18,461
SPIGa	SPIGa2012a	SPIGa2012a_metadata.csv	2419
	SPIGa2013b	SPIGa2013b_metadata.csv	12,435
	SPIGa2014	SPIGa2014a_metadata.csv	1658
		SPIGa2014b_metadata.csv	1235
List of ‘PW Anonymised Raw Classifications and Metadata’ files stored in the Dryad Digital Repository (Data Citation 1), and their associated cameras/image files. The number of unique *Penguin Watch* volunteers that contributed classifications to each file are also shown. Metadata are extracted using R code found on GitHub at https://github.com/zooniverse/Data-digging/blob/master/example_scripts/Penguin_Watch/Penguin_Watch_ImageProcessingScript.R and Figshare^19^ (static version).			
