# Author Correction: Time-lapse imagery and volunteer classifications from the Zooniverse Penguin Watch project

**DOI:** 10.1038/s41597-019-0165-8

**Published:** 2019-09-06

**Authors:** Fiona M. Jones, Campbell Allen, Carlos Arteta, Joan Arthur, Caitlin Black, Louise M. Emmerson, Robin Freeman, Greg Hines, Chris J. Lintott, Zuzana Macháčková, Grant Miller, Rob Simpson, Colin Southwell, Holly R. Torsey, Andrew Zisserman, Tom Hart

**Affiliations:** 1 Department of Zoology, University of Oxford, South Parks Road, Oxford, OX1 3PS UK; 2 Zooniverse, Department of Physics, University of Oxford, Denys Wilkinson Building, Keble Road, Oxford, OX1 3RH UK; 3 Department of Engineering Science, University of Oxford, Parks Road, Oxford, OX1 3PJ UK; 4 Department of the Environment and Energy, Australian Antarctic Division, 203 Channel Highway, Kingston, Tasmania 7050 Australia; 5 Institute of Zoology, Wellcome Building, Outer Circle, Regent’s Park, London, NW1 4RY UK; 6 Google UK, Belgrave House, 76 Buckingham Palace Road, London, SW1W 9TQ UK

Correction to: *Scientific Data* 10.1038/sdata.2018.124, published online 26 June 2018

Following publication, it was discovered that two of the datasets deposited to Dryad to accompany this Data Descriptor were duplicates. The affected folders were named PETEc2014 and PETEd2013. As a result of this, there are a number of instances in the text where the total number of included images, the number of cameras that they were captured by, and the number of metadata files is stated incorrectly. These errors and the corresponding correct values are listed below:

- In the Abstract and Data Records section it is incorrectly stated that images were captured from 15 cameras. The correct number is 14.
- In the Abstract, Data Records section and Figure 4 legend it is incorrectly stated that 73,802 images were captured in total. The correct number of images is 63,070.
- In Table 4 and its accompanying footnote it is also incorrectly stated that 73,802 images were captured in total. The correct number of images is 63,070.
- Tables 4, 5 and 6 mention the duplicate PETEd2013 folder. This has now been removed from the Dryad dataset.

- In the Data Records section it is incorrectly stated that images are grouped into 23 separate folders, with 34 corresponding ‘consensus click’ folders and 34 metadata folders. Since the PETEd2013 image folder and associated citizen science files have been removed, the correct number of image folders is 22, and there are 32 corresponding ‘consensus click’ and metadata files.

Please note that the PETEc images used for the Technical Validation were randomly selected from the PETEc2013 dataset (not PETEc2014), and the PETEd2013 citizen science classifications were not incorporated into this analysis.

A new version of the dataset has been deposited to Dryad, from which the duplicate image folder (PETEd2013) and citizen science data files (PETEd2013a_concl, PETEd2013b_concl, PETEd2013a_metadata and PETEd2013b_metadata) have been removed. An accompanying version history and Erratum notice have been added to account for the change. The new version can be accessed via the existing citation in the accompanying Data Descriptor, or directly at 10.5061/dryad.vv36g.2.

